# Relation of Pulse Pressure to Long-Distance Gait Speed in Community-Dwelling Older Adults: Findings from the LIFE-P Study

**DOI:** 10.1371/journal.pone.0049544

**Published:** 2012-11-21

**Authors:** Kevin S. Heffernan, Todd M. Manini, Fang-Chi Hsu, Steven N. Blair, Barbara J. Nicklas, Stephen B. Kritchevsky, Anne B. Newman, Kim Sutton-Tyrrell, Timothy S. Church, William L. Haskell, Roger A. Fielding

**Affiliations:** 1 Department of Exercise Science, Human Performance Laboratory, Syracuse University, Syracuse, New York, United States of America; 2 Department of Aging and Geriatric Research, University of Florida, Gainesville, Florida, United States of America; 3 Department of Biostatistical Sciences, Division of Public Health Sciences, Wake Forest University Health Sciences, Winston-Salem, North Carolina, United States of America; 4 Arnold School of Public Health, Department of Exercise Science, University of South Carolina, Columbia, South Carolina, United States of America; 5 J. Paul Sticht Center on Aging, Department of Internal Medicine, Wake Forest University School of Medicine, Winston-Salem, North Carolina, United States of America; 6 Center for Aging and Population Health, Department of Epidemiology, University of Pittsburgh, Pittsburgh, Pennsylvania, United States of America; 7 Pennington Biomedical Research Center, Louisiana State University System, Baton Rouge, Louisiana, United States of America; 8 Stanford Prevention Research Center, Stanford University School of Medicine, Stanford, California, United States of America; 9 Nutrition, Exercise Physiology and Sarcopenia Laboratory, Jean Mayer United States Department of Agriculture Human Nutrition Research Center on Aging at Tufts University, Boston, Massachusetts, United States of America; INRCA, Italy

## Abstract

**Background:**

Reduced gait speed is associated with falls, late-life disability, hospitalization/institutionalization and cardiovascular morbidity and mortality. Aging is also accompanied by a widening of pulse pressure (PP) that contributes to ventricular-vascular uncoupling. The purpose of this study was to test the hypothesis that PP is associated with long-distance gait speed in community-dwelling older adults in the Lifestyle Interventions and Independence for Elders Pilot (LIFE-P) study.

**Methods:**

Brachial blood pressure and 400-meter gait speed (average speed maintained over a 400-meter walk at “usual” pace) were assessed in 424 older adults between the ages of 70–89 yrs at risk for mobility disability (mean age = 77 yrs; 31% male). PP was calculated as systolic blood pressure (BP) – diastolic BP.

**Results:**

Patients with a history of heart failure and stroke (*n* = 42) were excluded leaving 382 participants for final analysis. When categorized into tertiles of PP, participants within the highest PP tertile had significantly slower gait speed than those within the lowest PP tertile (p<0.05). Following stepwise multiple regression, PP was significantly and inversely associated with 400-meter gait speed (p<0.05). Other significant predictors of gait speed included: handgrip strength, body weight, age and history of diabetes mellitus (p<0.05). Mean arterial pressure, systolic BP and diastolic BP were not predictors of gait speed.

**Conclusions:**

Pulse pressure is associated long-distance gait speed in community-dwelling older adults. Vascular senescence and altered ventricular-vascular coupling may be associated with the deterioration of mobility and physical function in older adults.

## Introduction

A critical factor in an older person’s ability to function independently is the ability to move without assistance. Older adults who lose mobility are less likely to remain in the community, have higher rates of mortality and experience a poorer quality of life [1], [2]. The 400-meter usual-pace walk test (400-MWT) is a highly reliable performance-based measure of mobility [3]. With advancing age, there is a general decline in gait speed. Reduced gait speed has been associated with falls, late-life disability, hospitalization and institutionalization [4]. Reduced gait speed has also been shown to be associated with several cardiovascular disease (CVD) risk factors [5]–[7], increased risk for ischemic stroke [8] and cardiovascular mortality [9]. Moreover, improving gait speed reduces mortality in older adults [10].

Blood pressure (BP) increases with advancing age, increasing cardiovascular morbidity and mortality. Changes in BP with aging exhibit marked heterogeneity as BP has distinct steady and pulsatile profiles. The steady component (i.e. mean arterial pressure, MAP) is largely influenced by cardiac output and peripheral vascular resistance. The pulsatile component (i.e. pulse pressure, PP) reflects the integration of left ventricular systolic function, large-artery stiffness/impedance, forward wave pressure and pressure from wave reflections. While mean arterial pressure changes little in older adulthood, PP can increase substantially owing to increases in arterial stiffness/impedance, genesis of a larger forward pressure wave, and faster arrival of larger reflected pressure waves [11]. In older adults, elevated PP increases risk for myocardial infarction [12], new-onset atrial fibrillation [13], heart failure [14] and is in an independent predictor of coronary disease [15] and cardiovascular mortality [16].

Several lines of investigation support a physiologic link between ventricular-vascular function (as appraised via pulse pressure) and physical function. Increased pulsatile pressure may reduce coronary perfusion, damage peripheral vessels reducing endothelial vasomotion and skeletal muscle perfusion, and reduce cerebral vasomotion creating white matter lesions in cortical regions of the brain integral in motor control. The summative effect would serve to alter gait performance. While high BP *per se* has been shown to be associated with reduced functional capacity [17], current gait speed [18], and longitudinal changes in gait speed over time [19], the relationship between absolute PP, as a proxy of ventricular-vascular function, and long-distance gait speed in older adults has not been specifically explored. The purpose of this study was to examine the association of the steady (MAP) and pulsatile (PP) components of BP with usual long distance gait speed measured during a 400-MWT in a large group of community-dwelling older adults at risk for mobility disability from The Lifestyle Interventions and Independence For Elders Pilot (LIFE-P) investigation. We hypothesized that elevated PP would be associated with lower gait speed in older adults with mobility limitations.

## Methods

### Ethics Statement

All participants provided written informed consent, and the institutional review boards of all participating institutions (Cooper Institute, Stanford University, University of Pittsburgh, and Wake Forest University) approved this study protocol. All aspects of this study were conducted in accordance with the principles expressed in the Declaration of Helsinki and is registered at http://www.ClinicalTrials.gov (registration # NCT00116194).

### Participants

The study participants consisted of 424 community-dwelling older adults between 70–89 years of age enrolled in the Lifestyle Intervention and Independence for Elders Pilot (LIFE-P) Study, a randomized controlled pilot clinical trial evaluating the effect of physical activity on mobility disability. Participants were included if they had functional limitations [defined as scoring ≤9 on the short physical performance battery [20]], were able to complete a 400-meter walk test within 15 minutes without the use of an assistive device, and had a sedentary lifestyle [defined as <20 minutes of regular physical activity per week during the prior month]. Other exclusion criteria included history of heart failure (New York Heart Association Class III or IV), stroke, aortic stenosis, uncontrolled angina, a Mini-Mental State Examination (MMSE) score less than 21, Parkinson’s disease, cancer requiring treatment in the past three years, and respiratory diseases necessitating regular use of corticosteroid pills/injections or the use of supplemental oxygen. Descriptive information on the cohort and study design for the LIFE-P trial has been previously described in detail [21], [22].

### Study Design

Short distance gait speed was measured as the time taken for participants to walk 4 meters at usual self-directed pace. Long distance gait speed was assessed by having individuals walk 10 laps at a comfortable, self-directed pace in a corridor between two cones spaced 20-m apart. Time to complete the 400-m walk was recorded in minutes and seconds. Gait speed was computed as time to complete the test divided by the distance. Participants were permitted to stop during the walk, but not allowed to sit or receive help from others (cane use was permitted during assessments). During the 400-MWT, any standing rest stop was allowed as long as it did not exceed 60 seconds.

Grip strength in both hands was measured using an adjustable, hydraulic grip strength dynamometer (Jamar Hydraulic Hand Dynamometer, Model No. BK-7498, Fred Sammons, Inc. Burr Ridge, IL) and taken as a proxy of overall muscular strength. Three trials were conducted for each hand and the averages of the left and right hand used for subsequent analyses.

Blood pressure (BP) was measured in duplicate using conventional auscultation and sphygmomanometry with participants in the seated position. Participants were instructed to remain in a fasted state, not consume alcohol, caffeine or perform heavy physical activity prior to blood pressure assessment. PP was calculated as systolic blood pressure (SBP) – diastolic blood pressure (DBP). MAP was calculated as (1/3 * SBP)+(2/3 * DBP). Heart rate (HR) was assessed in duplicate via palpation of the radial artery. The average of the two BP and HR measures were used for subsequent analyses. BP and HR assessment was carried out prior to walk testing and handgrip testing by trained study personnel.

### Statistical Analyses

All data are reported as means ± standard error of the mean (SEM). A priori significance was set at p<0.05 for a two sided test. Normality of distribution was assessed using Kolmogorov-Smirnov and Shapiro-Wilk tests. Participants were categorized into tertiles according to pulse pressure. Gait speed, along with other continuous variables, was compared across tertiles using ANOVA (Tukey post hoc comparisons). If group differences existed in potential confounders, these variables were entered into the model as covariates (ANCOVA). Chi-square tests were used to compare categorical variables across tertiles. Univariate associations were examined with Pearson’s correlation coefficients. Stepwise multiple regression was used to examine predictors of absolute 400-m gait speed. Variables entered into the model included: age, gender, grip strength, body weight, systolic blood pressure, diastolic blood pressure, mean arterial pressure, pulse pressure, heart rate, medication history (use of statins, aspirin, hormone replacement therapy, beta-blockers, angiotensin converting enzyme inhibitors/angiotensin receptor blockers, calcium channel blockers, diuretics), history of hypertension, diabetes mellitus, arthritis, myocardial infarction (stable coronary disease), smoking and clinic examination site. We then used the enter method to specifically compare the association of the individual BP components with gait speed. Those variables that previously demonstrated univariate associations with gait speed were first entered and they included: age, handgrip strength, body mass and presence of diabetes mellitus (p<0.1). Sex and heart rate were forced into the model. Separate models were then created by entering each BP variable (SBP, DBP, MAP, and PP) into a second block using a hierarchical design. A final model was created that adjusted for PP after inclusion of MAP with aforementioned co-variables. The R^2^ change and F change were computed to evaluate each model fit. Finally, participants with 400-meter gait speed <1.0 m/s were identified and defined as having slow gait speed according to a previously established clinical cut point [23]. Receiver operating characteristic (ROC) curves were generated to examine the sensitivity of PP and MAP to predict slow gait (as a dichotomous variable) in older adults. All data analysis was carried out using SPSS version 16.0 GP (SPSS, Inc., Chicago, IL).

## Results

Older adults (*n* = 424) between the ages of 70 and 89 with a short physical performance battery score ≤9 participated in this study. Patients with a history of heart failure and stroke (*n* = 42) were excluded from the present study due to the potential confounding influence of these conditions on 400-meter gait speed and/or pulse pressure. Thus 382 participants were included in the final analyses. By study design, all participants completed the 400-meter gait test.

Participants were categorized according to tertile of pulse pressure (Table 1). Participants within the highest pulse pressure tertile had significantly slower 400 m gait speed than those within the lowest pulse pressure tertile (Table 1, p<0.05). As also can be seen from Table 1, there were significant differences in systolic blood pressure, diastolic blood pressure, mean arterial pressure, heart rate, ACEi/ARB use and β -blocker use across tertiles (p<0.05). Adjusting for tertile differences in mean arterial pressure and/or ACEi/ARB use with ANCOVA had no effect on group differences in gait speed (adjusted means: 0.89 m/s; tertile 2, 0.86 m/s; tertile 3, 0.82 m/s; p = 0.011).

**Table 1:** Participant characteristics according to tertile of pulse pressure.

variable	All	Tertile 1	Tertile 2	Tertile 3
	*n* = 382	*n* = 126	*n* = 132	*n* = 124
Age, years	76.7±0.2	76.4±0.4	76.3±0.4	77.3±0.4
Male, %	29	33	29	25
Weight, kg	81.7±1.0	81.3±1.7	82.7±1.5	80.1±1.5
Smoker, %	3	3	2	4
SBP, mmHg	133±1	119±2	132±1†	147±2† ‡
DBP, mmHg	70±1	72±1	70±1	67±1† ‡
MAP, mmHg	91±1	88±1	91±1†	94±1† ‡
PP, mmHg	63±1	47±0.5	62±1†	80±1† ‡
HR, bpm	69±1	72±1	69±1	66±1†
400 m gait speed, m/s	0.86±0.01	0.89±0.02	0.86±0.02	0.82±0.01†
4 m gait speed, m/s	0.74±0.01	0.74±0.01	0.75±0.01	0.73±0.01
Handgrip strength, kg	25.0±0.5	25.3±0.8	25.4±0.8	24.1±0.7
Medical History, %				
Hypertension	68	61	68	73
Myocardial infarction	8	10	5	10
Diabetes mellitus	21	17	20	27
Osteoarthritis	23	21	27	20
Medications, %				
β-blocker	29	25	24	39† ‡
β_1_ Selective	24	22	20	32
Non-Selective	4	2	4	6
Calcium channel blocker	26	22	24	33
ACE/ARB	26	22‡	36	20‡
Diuretic	37	33	39	40
Statin	36	41	32	35
ASA	48	48	50	46
Hypoglycemic	17	12	16	23
Insulin	2	1	2	2
HRT	12	11	11	14

† Significantly different than Tertile 1 (p<0.05).

‡ Significantly different than Tertile 2 (p<0.05).

Data are mean+/−SEM.

Table 2 shows participant characteristics according to gait speed classification. Compared to older adults with gait speed ≥1.0 m/s, older adults with slow gait speed (defined as having gait speed <1.0 m/s; *n* = 297) were significantly older (p<0.05), had higher body mass (p<0.05), lower handgrip strength (p<0.05), higher prevalence of hypertension (p<0.05), greater use of calcium channel blockers (p<0.05) and a greater prevalence of diabetes mellitus (p<0.05). Older adults with slow gait speed also had significantly higher PP than older adults with gait speed ≥1.0 m/s (p<0.05). Differences in PP remained after adjusting for group differences in aforementioned variables (63.6±0.9 versus 59.2±1.9, p<0.05). ROC curve analysis revealed that PP added incremental value to slow gait prediction over that provided by age, sex, handgrip strength, body mass and presence of diabetes mellitus (AUC from 0.776 to 0.784). MAP did not improve the AUC (0.776).

**Table 2:** Participant characteristics according to slow gait speed (defined as <1.0 m/s).

variable	<1.0 m/s	≥1.0 m/s
	*n* = 297	*n* = 85
Age, years	77.2±0.2	74.7±0.4†
Male, %	27	36
Weight, kg	82.7±1.1	78.2±1.7†
Smoker, %	3	5
SBP, mmHg	133±1	131±2
DBP, mmHg	69±1	72±1†
MAP, mmHg	91±1	92±1
PP, mmHg	64±1	58±1†
HR, bpm	69±1	69±1
400 m gait speed, m/s	0.79±0.01	1.10±0.01†
4 m gait speed, m/s	0.71±0.01	0.83±0.01†
Handgrip strength, kg	24.1±0.5	28.3±0.8†
Medical History, %		
Hypertension	71	55†
Myocardial infarction	9	5
Diabetes mellitus	24	11
Osteoarthritis	23	20
Medications, %		
β-blocker	30	25
β_1_ Selective	26	21
Non-Selective	4	4
Calcium channel blocker	29	16†
ACE/ARB	27	24
Diuretic	38	35
Statin	35	41
ASA	50	41
Hypoglycemic	19	11
Insulin	2	1
HRT	11	16

† Significantly different than <1.0 m/s (p<0.05).

Data are mean+/−SEM.

As can be seen from Table 3, according to stepwise multiple regression, pulse pressure was a significant predictor of gait speed (p<0.05) as was handgrip strength (p<0.05), age (p<0.05), body weight (p<0.05), and history of diabetes mellitus (p<0.05). Overall, the model accounted for 24.6% of the variance in 400 m gait speed. SBP, DBP and MAP were not predictors of absolute gait speed according to multiple regression. There was no association between PP and 4 m gait speed (r = −0.04, p>0.05) and 4 m gait speed did not differ across tertiles of PP. When specifically comparing the separate BP components, PP was the only significant predictor of gait speed and remained significant after additionally adjusting for MAP (Table 4).

**Table 3:** *Predictors of 400-meter gait speed.

*Variable*	Standardized β	R^2^ Change	95% Confidence Interval	p-value
Handgrip strength	0.353	0.083	0.005–0.009	<0.001
Age	−0.307	0.055	−0.017– −0.009	<0.001
Weight	−0.293	0.079	−0.004– −0.002	<0.001
Diabetes mellitus	−0.124	0.017	−0.094– −0.014	0.008
Pulse pressure	−0.107	0.011	−0.002– −0.001	0.021

* only significant independent predictors of gait speed according to multiple regression.

**Table 4:** Multivariable adjusted* relations between BP components and gait speed.

*Variable*	Standardized β	R^2^	R^2^ Change	F Change	P-value
SBP	−0.084	0.244	0.007	3.252	0.072
DBP	0.017	0.237	0.000	0.131	0.718
MAP	−0.038	0.238	0.001	0.665	0.415
PP	−0.108	0.248	0.011	5.379	0.021
PP^a^	−0.109	0.248	0.010	4.694	0.031

* Adjusted for age, sex, heart rate, handgrip strength, diabetes, and body mass.

a Further adjusted for MAP.

To separately examine the effect of β-blocker use and heart rate on pulse pressure and gait speed, older adults were separated into 2 groups according to β-blocker use; those taking β-blockers (*n* = 124) versus not taking β-blockers (*n* = 258). Compared to older adults not taking the drug, those taking β-blockers had higher pulse pressure (66±2 versus 61±1 mmHg, p<0.05), lower heart rate (63±1versus 71±1 mmHg, p<0.05) and a trend toward lower gait speed (0.84±0.01 versus 0.87±0.01, m/s, p = 0.09). Adjusting for group differences in heart rate attenuated group differences in pulse pressure (adjusted means: 64±1 versus 62±1; p>0.05) and gait speed (adjusted means: 0.84±0.01 versus 0.86±0.01; p = 0.30). Overall, there was an inverse association between heart rate and pulse pressure (r = −0.22, p<0.05). There were no differences in PP (66±2 versus 67±4 mmHg, p>0.05) or gait speed (0.85±0.01 vs 0.87±0.05; p>0.05) when comparing hypertensive participants taking β_1_-selective agents versus non-selective agents.

Women had slower age- and heart-rate adjusted 400 m gait speed (0.85±0.01 versus 0.89±0.02, p = 0.037) and pulse pressure (60±1 versus 64±1 mmHg, p = 0.028) than men. When co-varying for PP with analysis of covariance, sex-differences in 400 m gait speed were attenuated (adjusted means: 0.86±0.01 versus 0.88±0.01; p = 0.10).

## Discussion

Older adults at risk for mobility disability comprise an ever growing proportion of the older adult population. This particular group is also at a higher risk for loss of independence, institutionalization, and death than older adults with higher physical function. Therefore understanding factors that may affect gait speed in older adults at risk for mobility disability is of significant clinical and practical concern. In support of previous work we noted several predictors of gait speed including age, body weight, handgrip strength (often used as a proxy of global muscular strength), and diabetes mellitus [24]. The novel aspect of the present investigation was that the pulsatile component of blood pressure (pulse pressure) was independently associated with long distance gait speed in community-dwelling older adults with mobility limitations, even after adjusting for other co-variables and the steady component of blood pressure (mean arterial pressure). Moreover, ROC curve analysis revealed that PP added incremental value to slow gait prediction (defined as gait speed <1.0 m/s) over that provided by age, body weight, muscular strength, and diabetes mellitus (AUC from 0.776 to 0.784).

PP is an easily obtainable measure of pulsatile afterload related to arterial stiffness, forward wave pressure and pressure from wave reflections. Brachial cuff-based measures of BP with subsequent calculation of PP do not require specialized equipment and are used in regular clinical practice giving this measure broad appeal. Elevated PP is associated with endothelial dysfunction [25], left ventricular hypertrophy [26], ischemia during exercise [27], and impaired ventricular relaxation [28], all clinically relevant facets of aging; all separately shown to be associated with reduced physical function. Our results build upon this and note that elevated PP is also an independent predictor of gait speed in older adults, a measure of physical function that in and of itself is a predictor of survival in older adults [29]. Older adults with higher PP had significantly slower gait speed compared to older adults with lower PP. These findings raise the intriguing possibility that age-associated decline in vascular function may be inextricably linked to decline in physical function.

In well-functioning older adults, PP/arterial stiffness may not be a predictor of short distance (2.4 m–20 m) gait speed [30], [31]. Our findings are consistent with this as we noted no association between PP and 4 m gait speed in older adults with mobility limitations from the LIFE-P cohort. We noted an association between long distance gait speed (400 m) and PP in older adults with mobility limitations. Recent work from the Health Aging and Body Composition (ABC) Study has noted that arterial stiffness is a predictor of gait speed in older adults with peripheral arterial disease (PAD) [30]. Previous studies have noted that arterial stiffness and pressure from wave reflections are also predictors of walking distance in patients with PAD [32]. Recently, our group has reported an association between PP and long-distance gait performance in adults with multiple sclerosis (MS) [33]. Mobility limitations, as seen with PAD and MS, perpetuate a sedentary lifestyle and physical inactivity is a potent instigator of vascular mal-adaptation (i.e. increased arterial stiffness, endothelial dysfunction, reduced limb blood flow). Older adults with mobility-limitations from LIFE-P with the slowest gait speed had substantially higher pulse pressure than older well-functioning adults from the Health ABC Study (80 mmHg vs. 68 mmHg) [34], suggesting hastened vascular senescence in the LIFE-P group. Thus our findings support previous conclusions that arterial stiffness may be especially detrimental to older adults with established compromised mobility and significantly impaired vascular function [30] and suggest that long distance gait performance but not short distance gait performance may be influenced by PP in older adults at risk for mobility disability.

Several potential mechanisms may explain the association between PP and gait performance. Left ventricular ejection of stroke volume into a stiff aorta (altered aortic impedance and subsequent forward wave genesis) coupled with early return of reflected pressure waves of greater magnitude increases cardiac energetic demand, reduces stroke volume (i.e. wave reflections augment pressure but subtract from flow), reduces myocardial oxygen supply/consumption and reduces subendocardial perfusion [35]. Pulsatile pressure and flow damages the endothelium which may alter oxygen delivery to and impair oxygen uptake by the working skeletal muscle [36]. Pulsatile pressure stemming from increased arterial stiffness is associated with retinal damage [37] and visual impairment is a predictor of disability and gait performance [38], [39]. Finally, pulsatile load may damage cerebral blood vessels, reduce cerebrovascular reactivity, and contribute to cerebral white matter hyperintensities [40] and cognitive decline [41]. Indeed white matter lesions may be an intermediate factor in the relation of hypertension and lower gait speed in older adults [18], [42] and cognitive function is associated with physical function [43].

Older adults taking beta-blockers had higher PP and a trend toward lower gait speed than older adults not taking these agents. This appears to have been mediated by the secondary effect of beta-blockers on heart rate as heart rate was significantly lower in those taking beta-blockers versus those not taking these agents. Adjusting for heart rate abolished differences in PP and gait speed. Reductions in heart rate with beta-blocker use may alter pressure wave temporal associations, increasing late systolic pressure augmentation [44] and widening PP. Moreover, increased arterial stiffness, as occurs with natural aging, may exacerbate the influence of HR on wave reflections [45]. Thus, therapies that negatively influence pressure from wave reflections and increase PP may have a detrimental effect on physical function in older adults with low already low vascular compliance. Additional research is needed to test this hypothesis empirically.

Women had slower 400 m gait speed and this is consistent with previous reports [46], [47]. However, sex was not a predictor of gait speed in LIFE-P. A reason for this may be related to concomitant sex-differences in PP. Women had higher PP than men in LIFE-P and this is also well established in the literature [48], [49]. It is speculated that due to shorter stature and hormonally mediated changes in vascular function, older women have increased arterial stiffness and augmented pressure from wave reflections contributing to higher PP. Interestingly, after adjusting for sex-differences in PP, there were no longer sex-differences in gait speed. Therefore, PP may offer physiologic insight into sex-differences in gait speed in older adults.

Limitations to this study should be noted. Presence or absence of PAD was not assessed in LIFE-P. Thus, it is possible that the association between PP and gait speed in LIFE-P was driven in part by the confounding influence of PAD, as previously reported in the Health ABC Study. Self-reports of leg pain during the 400 m walk test were not high in LIFE-P (*n* = 16) and participants reporting leg pain had similar PP as those participants not reporting leg pain (64 mmHg vs. 62 mmHg, p = 0.6). A specific inclusion criterion for the LIFE-P, and novel aspect of the present cohort, was presence of functional limitation. Thus, present findings may not be extrapolated to older adults with higher functional capacity. The main focus for this study was the exploration of PP as a physiologic correlate of gait. In unadjusted models, PP accounted for 2% of the variance in 400 m gait speed. Although modest, PP was able to improve prediction of slow gate speed using ROC analysis. Future research that appraises clinical outcomes with measures of gait speed and PP are needed to examine the clinical implications of present findings using proper calculation of net reclassification improvement.

In conclusion, PP is a predictor of gait speed in community-dwelling older adults. Although noted associations are modest, these findings support that vascular senescence and altered ventricular-vascular coupling may contribute, in part, to the deterioration of physical function with advancing age. Future research is needed to examine whether therapeutic interventions that specifically target PP (and not SBP or DBP *per se*) have clinical utility as a means of improving physical function with advancing age.

## References

[1] Hirvensalo M, Rantanen T, Heikkinen E (2000) Mobility difficulties and physical activity as predictors of mortality and loss of independence in the community-living older population. J Am Geriatr Soc 48: 493–498. 10811541 10.1111/j.1532-5415.2000.tb04994.x

[2] Guralnik JM, LaCroix AZ, Branch LG, Kasl SV, Wallace RB (1991) Morbidity and disability in older persons in the years prior to death. Am J Public Health 81: 443–447. 2003621 10.2105/ajph.81.4.443 PMC1405038

[3] Studenski S (2009) Bradypedia: is gait speed ready for clinical use? J Nutr Health Aging 13: 878–880. 19924347 10.1007/s12603-009-0245-0 PMC12878062

[4] Abellan van Kan G, Rolland Y, Andrieu S, Bauer J, Beauchet O, et al (2009) Gait speed at usual pace as a predictor of adverse outcomes in community-dwelling older people an International Academy on Nutrition and Aging (IANA) Task Force. J Nutr Health Aging 13: 881–889. 19924348 10.1007/s12603-009-0246-z PMC12878092

[5] Elbaz A, Ripert M, Tavernier B, Fevrier B, Zureik M, et al (2005) Common carotid artery intima-media thickness, carotid plaques, and walking speed. Stroke 36: 2198–2202. 16166578 10.1161/01.STR.0000181752.16915.5c

[6] Inzitari M, Naydeck BL, Newman AB (2008) Coronary artery calcium and physical function in older adults: the Cardiovascular Health Study. J Gerontol A Biol Sci Med Sci 63: 1112–1118. 18948563 10.1093/gerona/63.10.1112 PMC4886308

[7] Kuo CK, Lin LY, Yu YH, Wu KH, Kuo HK (2009) Inverse association between insulin resistance and gait speed in nondiabetic older men: results from the U.S. National Health and Nutrition Examination Survey (NHANES) 1999–2002. BMC Geriatr 9: 49. 19922671 10.1186/1471-2318-9-49 PMC2784762

[8] McGinn AP, Kaplan RC, Verghese J, Rosenbaum DM, Psaty BM, et al (2008) Walking speed and risk of incident ischemic stroke among postmenopausal women. Stroke 39: 1233–1239. 18292379 10.1161/STROKEAHA.107.500850

[9] Dumurgier J, Elbaz A, Ducimetiere P, Tavernier B, Alperovitch A, et al (2009) Slow walking speed and cardiovascular death in well functioning older adults: prospective cohort study. BMJ 339: b4460. 19903980 10.1136/bmj.b4460 PMC2776130

[10] Hardy SE, Perera S, Roumani YF, Chandler JM, Studenski SA (2007) Improvement in usual gait speed predicts better survival in older adults. J Am Geriatr Soc 55: 1727–1734. 17916121 10.1111/j.1532-5415.2007.01413.x

[11] Mitchell GF, Wang N, Palmisano JN, Larson MG, Hamburg NM, et al (2010) Hemodynamic correlates of blood pressure across the adult age spectrum: noninvasive evaluation in the framingham heart study. Circulation 122: 1379–1386. 20855656 10.1161/CIRCULATIONAHA.109.914507 PMC2981604

[12] Vaccarino V, Holford TR, Krumholz HM (2000) Pulse pressure and risk for myocardial infarction and heart failure in the elderly. J Am Coll Cardiol 36: 130–138. 10898424 10.1016/s0735-1097(00)00687-2

[13] Mitchell GF, Vasan RS, Keyes MJ, Parise H, Wang TJ, et al (2007) Pulse pressure and risk of new-onset atrial fibrillation. JAMA 297: 709–715. 17312290 10.1001/jama.297.7.709

[14] Chae CU, Pfeffer MA, Glynn RJ, Mitchell GF, Taylor JO, et al (1999) Increased pulse pressure and risk of heart failure in the elderly. JAMA 281: 634–639. 10029125 10.1001/jama.281.7.634

[15] Franklin SS, Larson MG, Khan SA, Wong ND, Leip EP, et al (2001) Does the relation of blood pressure to coronary heart disease risk change with aging? The Framingham Heart Study. Circulation 103: 1245–1249. 11238268 10.1161/01.cir.103.9.1245

[16] Domanski M, Norman J, Wolz M, Mitchell G, Pfeffer M (2001) Cardiovascular risk assessment using pulse pressure in the first national health and nutrition examination survey (NHANES I). Hypertension 38: 793–797. 11641288 10.1161/hy1001.092966

[17] Hajjar I, Lackland DT, Cupples LA, Lipsitz LA (2007) Association between concurrent and remote blood pressure and disability in older adults. Hypertension 50: 1026–1032. 18025294 10.1161/HYPERTENSIONAHA.107.097667 PMC2424316

[18] Dumurgier J, Elbaz A, Dufouil C, Tavernier B, Tzourio C (2010) Hypertension and lower walking speed in the elderly: the Three-City study. J Hypertens 28: 1506–1514. 20404744 10.1097/HJH.0b013e328338bbec PMC4851985

[19] Rosano C, Longstreth WT Jr, Boudreau R, Taylor CA, Du Y, et al (2011) High blood pressure accelerates gait slowing in well-functioning older adults over 18-years of follow-up. J Am Geriatr Soc 59: 390–397. 21391929 10.1111/j.1532-5415.2010.03282.x PMC3637929

[20] Guralnik JM, Simonsick EM, Ferrucci L, Glynn RJ, Berkman LF, et al (1994) A short physical performance battery assessing lower extremity function: association with self-reported disability and prediction of mortality and nursing home admission. J Gerontol 49: M85–94. 8126356 10.1093/geronj/49.2.m85

[21] Katula JA, Kritchevsky SB, Guralnik JM, Glynn NW, Pruitt L, et al (2007) Lifestyle Interventions and Independence for Elders pilot study: recruitment and baseline characteristics. J Am Geriatr Soc 55: 674–683. 17493186 10.1111/j.1532-5415.2007.01136.x

[22] Rejeski WJ, Fielding RA, Blair SN, Guralnik JM, Gill TM, et al (2005) The lifestyle interventions and independence for elders (LIFE) pilot study: design and methods. Contemp Clin Trials 26: 141–154. 15837437 10.1016/j.cct.2004.12.005

[23] Simonsick EM, Newman AB, Visser M, Goodpaster B, Kritchevsky SB, et al (2008) Mobility limitation in self-described well-functioning older adults: importance of endurance walk testing. J Gerontol A Biol Sci Med Sci 63: 841–847. 18772472 10.1093/gerona/63.8.841 PMC5722013

[24] Brach JS, Talkowski JB, Strotmeyer ES, Newman AB (2008) Diabetes mellitus and gait dysfunction: possible explanatory factors. Phys Ther 88: 1365–1374. 18801861 10.2522/ptj.20080016 PMC2579906

[25] Kuvin JT, Sidhu M, Patel AR, Sliney KA, Pandian NG, et al (2005) Pulse pressure and peripheral arterial vasoreactivity. J Hum Hypertens 19: 501–502. 15729377 10.1038/sj.jhh.1001844

[26] Gardin JM, Arnold A, Gottdiener JS, Wong ND, Fried LP, et al (1997) Left ventricular mass in the elderly. The Cardiovascular Health Study. Hypertension 29: 1095–1103. 9149672 10.1161/01.hyp.29.5.1095

[27] Kingwell BA, Waddell TK, Medley TL, Cameron JD, Dart AM (2002) Large artery stiffness predicts ischemic threshold in patients with coronary artery disease. J Am Coll Cardiol 40: 773–779. 12204510 10.1016/s0735-1097(02)02009-0

[28] Leite-Moreira AF, Correia-Pinto J, Gillebert TC (1999) Afterload induced changes in myocardial relaxation: a mechanism for diastolic dysfunction. Cardiovasc Res 43: 344–353. 10536664 10.1016/s0008-6363(99)00099-1

[29] Studenski S, Perera S, Patel K, Rosano C, Faulkner K, et al (2011) Gait speed and survival in older adults. JAMA 305: 50–58. 21205966 10.1001/jama.2010.1923 PMC3080184

[30] Watson NL, Sutton-Tyrrell K, Youk AO, Boudreau RM, Mackey RH, et al (2011) Arterial stiffness and gait speed in older adults with and without peripheral arterial disease. Am J Hypertens 24: 90–95. 20940711 10.1038/ajh.2010.193 PMC3290653

[31] Brunner EJ, Shipley MJ, Witte DR, Singh-Manoux A, Britton AR, et al (2011) Arterial stiffness, physical function, and functional limitation: the Whitehall II Study. Hypertension 57: 1003–1009. 21444833 10.1161/HYPERTENSIONAHA.110.168864 PMC6532973

[32] Brewer LC, Chai HS, Bailey KR, Kullo IJ (2007) Measures of arterial stiffness and wave reflection are associated with walking distance in patients with peripheral arterial disease. Atherosclerosis 191: 384–390. 16730015 10.1016/j.atherosclerosis.2006.03.038

[33] Heffernan KS, Ranadive S, Weikert M, Lane A, Yan H, et al (2011) Pulse pressure is associated with walking impairment in multiple sclerosis. J Neurol Sci 309: 105–109. 21821264 10.1016/j.jns.2011.07.004

[34] Watson NL, Sutton-Tyrrell K, Youk AO, Boudreau RM, Mackey RH, et al.. (2011) Arterial Stiffness and Gait Speed in Older Adults With and Without Peripheral Arterial Disease. Am J Hypertens. 10.1038/ajh.2010.193 PMC3290653 20940711

[35] Saeki A, Recchia F, Kass DA (1995) systolic flow augmentation in hearts ejecting into a model of stiff aging vasculature. Influence on myocardial perfusion-demand balance. Circ Res 76: 132–141. 8001271 10.1161/01.res.76.1.132

[36] Poole DC, Musch TI (2010) Muscle microcirculatory O(2) exchange in health and disease. Adv Exp Med Biol 662: 301–307. 20204807 10.1007/978-1-4419-1241-1_43

[37] Katsi V, Vlachopoulos C, Souretis G, Baou K, Dagalaki I, et al (2012) Association between retinal microcirculation and aortic stiffness in hypertensive patients. Int J Cardiol 157: 370–373. 21256605 10.1016/j.ijcard.2010.12.074

[38] Cacciatore F, Abete P, Maggi S, Luchetti G, Calabrese C, et al (2004) Disability and 6-year mortality in elderly population. Role of visual impairment. Aging Clin Exp Res 16: 382–388. 15636464 10.1007/BF03324568

[39] Kim DH, Chaves PH, Newman AB, Klein R, Sarnak MJ, et al (2012) Retinal microvascular signs and disability in the Cardiovascular Health Study. Arch Ophthalmol 130: 350–356. 22084159 10.1001/archophthalmol.2011.360 PMC3520093

[40] Shrestha I, Takahashi T, Nomura E, Ohtsuki T, Ohshita T, et al (2009) Association between central systolic blood pressure, white matter lesions in cerebral MRI and carotid atherosclerosis. Hypertens Res 32: 869–874. 19644503 10.1038/hr.2009.121

[41] Mitchell GF, van Buchem MA, Sigurdsson S, Gotal JD, Jonsdottir MK, et al (2011) Arterial stiffness, pressure and flow pulsatility and brain structure and function: the Age, Gene/Environment Susceptibility–Reykjavik study. Brain 134: 3398–3407. 22075523 10.1093/brain/awr253 PMC3212721

[42] Hajjar I, Quach L, Yang F, Chaves PH, Newman AB, et al (2011) Hypertension, white matter hyperintensities, and concurrent impairments in mobility, cognition, and mood: the Cardiovascular Health Study. Circulation 123: 858–865. 21321150 10.1161/CIRCULATIONAHA.110.978114 PMC3081662

[43] Atkinson HH, Rosano C, Simonsick EM, Williamson JD, Davis C, et al (2007) Cognitive function, gait speed decline, and comorbidities: the health, aging and body composition study. J Gerontol A Biol Sci Med Sci 62: 844–850. 17702875 10.1093/gerona/62.8.844

[44] Heffernan KS, Suryadevara R, Patvardhan EA, Mooney P, Karas RH, et al (2011) Effect of atenolol vs metoprolol succinate on vascular function in patients with hypertension. Clin Cardiol 34: 39–44. 21259277 10.1002/clc.20841 PMC6652434

[45] Papaioannou TG, Vlachopoulos CV, Alexopoulos NA, Dima I, Pietri PG, et al (2008) The effect of heart rate on wave reflections may be determined by the level of aortic stiffness: clinical and technical implications. Am J Hypertens 21: 334–340. 18219305 10.1038/ajh.2007.52

[46] Samson MM, Crowe A, de Vreede PL, Dessens JA, Duursma SA, et al (2001) Differences in gait parameters at a preferred walking speed in healthy subjects due to age, height and body weight. Aging (Milano) 13: 16–21. 11292147 10.1007/BF03351489

[47] Callisaya ML, Blizzard L, Schmidt MD, McGinley JL, Srikanth VK (2008) Sex modifies the relationship between age and gait: a population-based study of older adults. J Gerontol A Biol Sci Med Sci 63: 165–170. 18314452 10.1093/gerona/63.2.165

[48] Segers P, Rietzschel ER, De Buyzere ML, Vermeersch SJ, De Bacquer D, et al (2007) Noninvasive (input) impedance, pulse wave velocity, and wave reflection in healthy middle-aged men and women. Hypertension 49: 1248–1255. 17404183 10.1161/HYPERTENSIONAHA.106.085480

[49] Franklin SS, Gustin Wt, Wong ND, Larson MG, Weber MA, et al (1997) Hemodynamic patterns of age-related changes in blood pressure. The Framingham Heart Study. Circulation 96: 308–315. 9236450 10.1161/01.cir.96.1.308

